# Zinc mediates the neuronal activity–dependent anti-apoptotic effect

**DOI:** 10.1371/journal.pone.0182150

**Published:** 2017-08-07

**Authors:** Mei Qiu, Yang-ping Shentu, Ji Zeng, Xiao-chuan Wang, Xiong Yan, Xin-wen Zhou, Xiao-peng Jing, Qun Wang, Heng-ye Man, Jian-zhi Wang, Rong Liu

**Affiliations:** 1 Department of Pathophysiology, Key Laboratory of Ministry of Education for Neurological Disorders, School of Basic Medicine, Tongji Medical College, Huazhong University of Science and Technology, Wuhan, China; 2 Clinical College, Hubei University of TCM, Wuhan, China; 3 Department of Clinic Laboratory, Pu Ai Hospital, Tongji Medical College, Huazhong University of Science and Technology, Wuhan, China; 4 Department of Biology, Boston University, Boston, Massachusetts, United States of America; Cleveland Clinic, UNITED STATES

## Abstract

Synaptic activity increases the resistance of neurons to diverse apoptotic insults; however, the underlying mechanisms remain less well understood. Zinc promotes cell survival under varied conditions, but the role of synaptically released zinc in the activity-dependent anti-apoptotic effect is unknown. Using cultured hippocampal slices and primary neurons we show that a typical apoptosis inducer–staurosporine (STP) was able to cause concentration-dependent apoptotic cell death in brain slices; Enhanced synaptic activity by bicuculline (Bic)/4-Aminopyridine (AP) treatment effectively prevented neurons from STP-induced cell apoptosis, as indicated by increased cell survival and suppressed caspase-3 activity. Application of Ca-EDTA, a cell membrane-impermeable zinc chelator which can efficiently capture the synaptically released zinc, completely blocked the neuronal activity-dependent anti-apoptotic effect. Same results were also observed in cultured primary hippocampal neurons. Therefore, our results indicate that synaptic activity improves neuronal resistance to apoptosis via synaptically released zinc.

## Introduction

During development, neurons with efficient synaptic transmission often survive, whereas those without functional connection will selectively undergo apoptotic cell death [1–2]. Consistently, mature neurons which have higher levels of synaptic activities show strong resistance to apoptosis-inducing factors [3]; while deprivation of neuronal activity induces cell apoptosis [4]. However, the mechanism underlying the anti-apoptotic effect of synaptic activity has not been fully understood.

The glutamate neurons are the major excitatory neurons in the human brain. Of the studies focusing on the protective effects of synaptic activity, most data was obtained from glutamatergic neurons [3, 5–6]. Zinc is a trace element which is enriched in the brain. About half of glutamatergic neurons are “gluzinergic” neurons. These neurons contain a large amount of zinc ions in the presynaptic vesicles, which is released into the synaptic cleft together with glutamate upon synaptic activity [7–10]. The released zinc thus acts as an important neuromodulator to regulate the glutamate receptor activities [11–13]. Zinc is present at high concentrations in the synaptic vesicles of hippocampal mossy fibers [14], synaptic zinc release from hippocampal slices upon increased neuronal activity induced by electric or chemical stimulation has been identified by different research groups [7,9,15–17].

A large amount of studies have shown that zinc deficiency or intracellular zinc depletion leads to neuronal apoptosis [18–19], while addition of zinc inhibits cell apoptosis [20–21]. We therefore suspect that synaptically released zinc from gluzinergic neurons may play a role in the anti-apoptotic effect under increased synaptic activation.

In the present study, we used cultured brain slices and primary neurons to explore the effects of synaptically released zinc in neuronal apoptosis. The results showed that synaptic activity dramatically reduced apoptosis inducer-caused neuronal death, and this effect was mediated by synaptically released zinc.

## Materials and methods

### Antibodies and reagents

Rabbit polyclonal antibodies (pAb) against PARP and cleaved caspase-3, mouse monoclonal antibody (mAb) against caspase-3 and apoptosis inducer staurosporine (STP) were all from Cell Signaling Technology (Danvers, MA, USA); mAb against NeuN and Cytochrome C were from Millipore (Billerica, MA, USA); mAb of DM1A, Ca-EDTA (1 mM), Bic (50 μM) and 4-AP (250 μM) were from Sigma (St. Louis, MO, USA). LDH assay kit was purchased from Promega (St. Madison, FL, USA).

### Brain slices culture

Adult male Sprague Dawley rats (male, 250–300 g) were from the Experimental Animal Center of Tongji Medical College, Huazhong University of Science and Technology. All animal experiments were approved by the Animal Care and Use Committee of Tongji Medical College, Huazhong University of Science and Technology. Rats were decapitated under anesthesia with isoflurane, the brains were rapidly removed and put into oxygenated (95% O_2_, 5% CO_2_) artificial cerebrospinal fluid (aCSF) containing 12 mM NaCl, 3.5 mM KCl, 1.2 mM NaH_2_PO_4_, 1.3 mM MgCl_2_, 2.0 mM CaCl_2_, 11 mM D(+)-glucose and 25 mM NaHCO_3_, pH 7.4, for 7–8 min at 4°C. Hippocampus were separated and mounted on a vibratome holder. Coronal hippocampal slices (400 μm thick) were sectioned with a Mcllwain Tissue Chopper (The Mickle Laboratory Engineering Co. Ltd, Gomshall, Surrey, UK). After 30 min equilibration at room temperature, the brain slices were transferred into incubating charmbers with aCSF bubbled with 95% O2 and 5% CO2 at 37°C for different treatments. For the study on the effect of synaptically zinc in the apoptosis, slices were incubated with or without Ca-EDTA (1 mM) for 30 min. Then slices were treated with STP (1 μM) for 3 h, with or without the pre-treatment of Bic (50 μM) and 4-AP (250 μM) to increase the synaptic activity for 2.5 h. At the end of treatment, the aCSF was collected for cytotoxicity assay by detecting the LDH levels. Slices were collected and homogenated for Western blotting detection of the apoptosis related proteins.

### Primary neuron culture

Primary hippocampal neurons were isolated from embryonic E18 Sprague Dawley rats. Briefly, embryonic hippocampus were dissected, dissociated, and incubated with 4 ml D-Hanks containing 0.125% trypsin for 15 min at 37°C, centrifuged at 1000 g for 5 min after addition of 4 ml of the neuronal plating medium containing DMEM/F12 with 10% fetal bovine serum, then the cells were resuspended and plated onto 12-cell plates for Western blotting or glass coverslips for cell imaging. Both the plates and the glass coverslips were previously coated with poly-D-lysine. The neurons were then put into a humidified incubator with 5% CO_2_ at 37°C. The medium was changed to Neurobasal medium supplemented with 2% B27 (maintenance medium) after 2–4 h. The cells were cultured for 9–11 days for treatment. During the culture, the medium was half-changed every 3 days with fresh maintenance medium. Hippocampal neurons were incubated with or without Ca-EDTA (1 mM) for 30 min. Then neurons were treated with STP (100 nM) for 24 h, with or without the pre-treatment of Bic (50 μM) and 4-AP (250 μM) for 16 h. At the end of treatment, cytotoxicity was measured by LDH assay; cell apoptosis related proteins were detected by Western blotting and immunofluorescence.

### Western blotting

Western blotting was performed according to the method established in our laboratory. Briefly, the cell lysates or brain tissue homogenates were mixed with sample buffer containing 50 mM Tris-HCl (pH 7.6), 2% SDS, 10% glycerol, 1 mM DTT, and 0.2% bromophenol blue and boiled for 5 min. The proteins were separated by 10% SDS/PAGE and transferred to PVDF membrane. The membranes were then blocked with 5% nonfat milk dissolved in TBSTween-20 (50 mM Tris-HCl, pH 7.6, 150 mM NaCl, 0.2% Tween-20) for 1 h and probed with primary antibody at 4°C overnight. Then the blots were detected by using anti-rabbit or anti-mouse IgG conjugated to IRDye (800CW; Licor Biosciences, Lincoln, NE,USA) for 1 h at room temperature and visualized using the Odyssey Infrared Imaging System (LicorBiosciences, Lincoln, NE, USA). The protein bands were quantitatively analyzed by odyssey software.

### LDH assay

The LDH assay was performed using a LDH cytotoxicity assay kit (Promega, Madison, FL, USA) according to the manufacturer's instructions. Briefly, aCSF from brain slices culture or culture media from primary hippocampal neuronal cultures (50 μl per sample) was mixed with assay buffers (50 μl) in wells of 96-well plate and incubated for 30 min at room temperature in darkness, then 50 μl stopping buffer were added to every well, the OD values were detected by microplate reader at 490 nm. Cytotoxicity = (Experimental group-Blank group)/ (Positive control group-Blank group) X100%.

### Immunofluorescence staining

The neurons were washed by PBS and fixed by 4% paraformaldehyde for 15 min, permeabilized in 0.1% Triton-X 100 for 15 min, followed by incubation with 3% bovine serum albumin (BSA) to block nonspecific sites. Subsequently, primary antibody against cleaved caspase-3 (1:200) and NeuN (1:200) incubation was performed overnight at 4°C. Cells were then washed by PBS for three times, followed by 1 h incubation with the secondary antibodies (1:200, A-21206, Invitrogen; A-10036, Invitrogen). At the end of incubation, cells were further incubated with Hoechst (1:1000) for 5 min, washed by PBS for three times. The coverslips were then sealed by 50% glycerol-PBS for observation under the LSM710 confocal microscope (Zeiss, Germany).

### Detection of zinc concentration in the aCSF

The zinc concentration in the aCSF was analyzed by atomic absorption spectrophotometry. At the end of brain slices incubation, 3 ml aCSF was collected for each sample, three replicates 1 ml per sample were analyzed using atomic absorption spectrophotometer AA-240FS from Varian (Palo Alto, California, USA).

#### Statistic analysis

Data are expressed as mean ± SEM, and analyzed using SPSS 16.0 statistical software (SPSS Inc., Chicago, IL, USA). The one-way analysis of variance (ANOVA) procedure followed by LSD’s post hoc tests was used to determine the differences among groups. All results shown correspond to individual representative experiments. The level of significance for all analysis was set at *p* < 0.05.

## Results

### Synaptic activity protects neurons against STP-induced apoptosis in rat hippocampal slices

**Fig 1:**
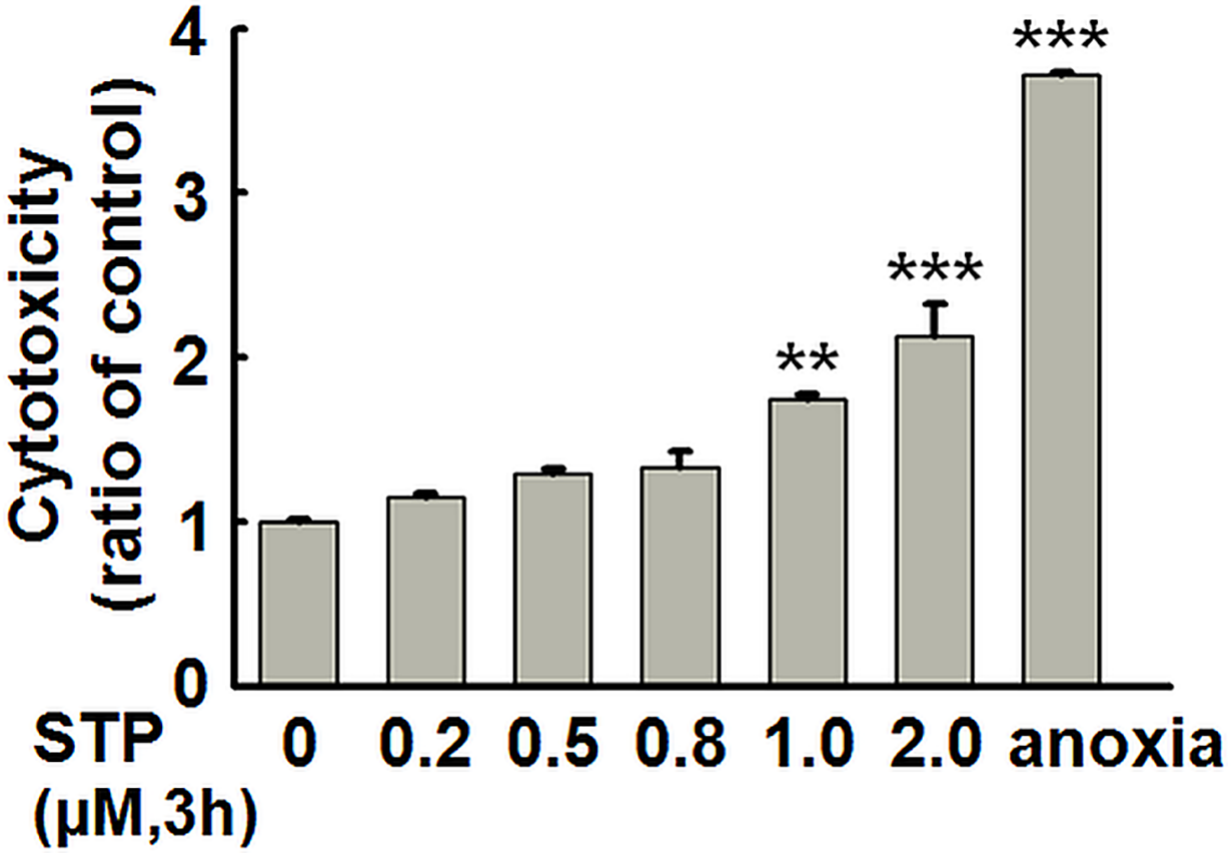
STP induces cytotoxicity in cultured rat hippocampal slices. Rat hippocampal slices were incubated with 0, 0.2, 0.5, 0.8, 1.0 or 2.0 μM STP for 3 h, the aCSF was collected and LDH levels were detected, slices cultured without oxygen supply were used as a positive control. aCSF: artificial cerebrospinal fluid. ** p<0.*01*, *** p<0.*001* versus control group. (n = 3).

**Fig 2:**
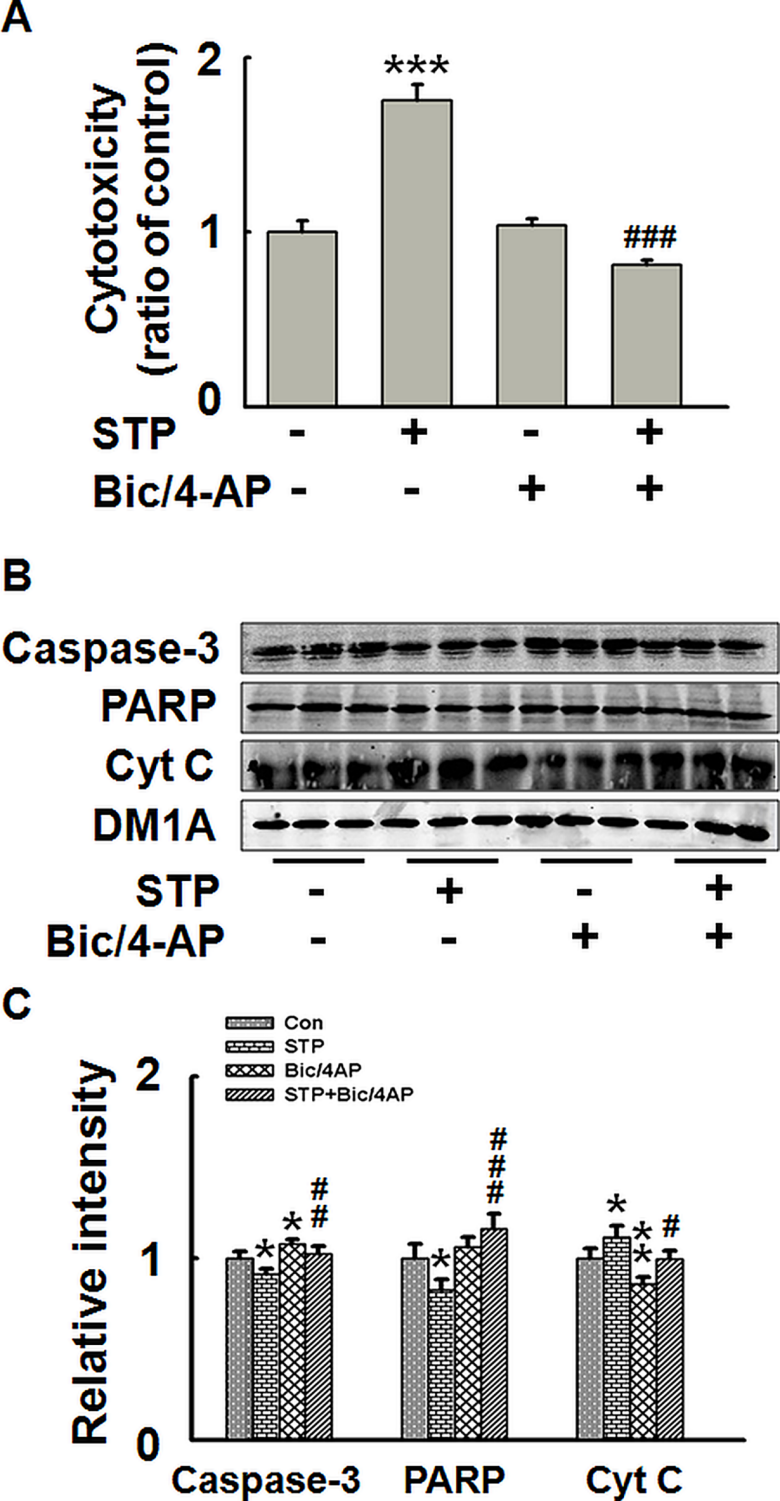
Increased synaptic activity protects neurons against apoptosis in rat hippocampal slices. Rat hippocampal slices were incubated with STP (1 μM) for 3 h, with or without the pre-treatment of GABA_A_ receptor antagonist Bic (50 μM) and potassium channels blocker 4-AP (250 μM) for 2.5 h. (A) LDH levels in the aCSF. *** p<0.*001* versus untreated control slices. *### p<0*.*001* versus STP-treated slices. (B) Apoptosis related proteins were detected by Western blotting. (C) Quantitative analysis of blots in (B) * p<0.*05*, ** p<0.*01* versus untreated control slices. *# p<0*.*05*, *##*, *p<0*.*01*,*### p<0*.*001* versus STP-treated slices. (n = 4).

STP is a commonly used apoptosis inducer. To explore the effect of synaptically released zinc in the positive effect of neuronal activity on cell survival, we first explored the effectiveness of STP in apoptosis induction in cultured hippocampal slices. The brain slices were treated with different concentrations of STP (0.2, 0.5, 0.8, 1.0 and 2.0 μM) for 3 hours, and then the cytotoxicity was measured through LDH assay. We found that STP in a concentration higher than 1.0 μM led to cell toxicity (Fig 1). We then used 1.0 μM STP to induce apoptosis in brain slices, with or without the incubation of Bic and 4-AP. Bic is a GABA_A_ receptor antagonist and 4-AP is a weak potassium ion channels blocker. Bic/4-AP treatment results in synchronized bursts of action potentials, which is a widely used model for enhanced synaptic activity [17]. As shown in Fig 2A, STP treatment induced significant cell death. Bic/4-AP treatment could reverse the STP-induced cytotoxicity to the control level. As a control, Bic/4-AP treatment itself had no effect on cell viability. These results confirmed the anti-apoptotic effect of synaptic activity. Detection of apoptosis related proteins showed that STP treatment resulted in decreased levels of uncleaved (inactivated) caspase-3 and uncleaved PARP, indicating caspase-3 activation. Again, Bic/4-AP treatment could reverse these changes (Fig 2B and 2C). Detection of cytochrome C (Cyt C) levels confirmed the STP-induced apoptosis and the prevention effect of Bic/4-AP treatment (Fig 2B and 2C). These data indicate that synaptic activity can prevent neurons from STP-induced apoptotic cell death in hippocampal slices.

### Exogeneous zinc protects against STP-induced apoptosis in rat hippocampal slices

**Fig 3:**
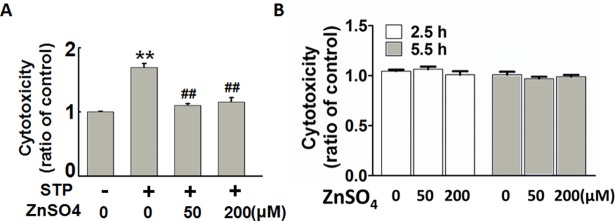
Exogeneous zinc protects rat hippocampal slices from STP-induced apoptosis. (A) Rat hippocampal slices were incubated with STP (1 μM) for 3 h, with or without the pre-treatment of 50 μM or 200 μM ZnSO_4_ for 2.5 h. (B) Rat hippocampal slices were incubated with 50 μM or 200 μM ZnSO_4_ for 2.5 h or 5.5 h, LDH levels in the aCSF were assayed. ** p<0.*01* versus untreated control slices. *## p<0*.*01* versus STP-treated slices. (n = 3).

Zinc is an indispensable trace element in human body. Previous studies have shown that neuronal apoptosis is induced when intracellular zinc is chelated or in condition of zinc deficiency [18–19]. To explore whether zinc has an anti-apoptotic effect in the present experimental system, we induced neuronal apoptosis with STP in the brain slices, with or without pre-incubation of ZnSO4(50 μM or 200 μM) for 2.5 h. The result showed that exogenous zinc could effectively protect cells from STP-induced cytotoxicity, as indicated by LDH release (Fig 3A); ZnSO_4_ incubation alone did not influence the cell viability (Fig 3B).Thus, exogeneous zinc protects against STP-induced apoptosis in rat hippocampal slices.

### Synaptically released zinc mediates the anti-apoptosis effect of synaptic activity in hippocampal slices

**Fig 4:**
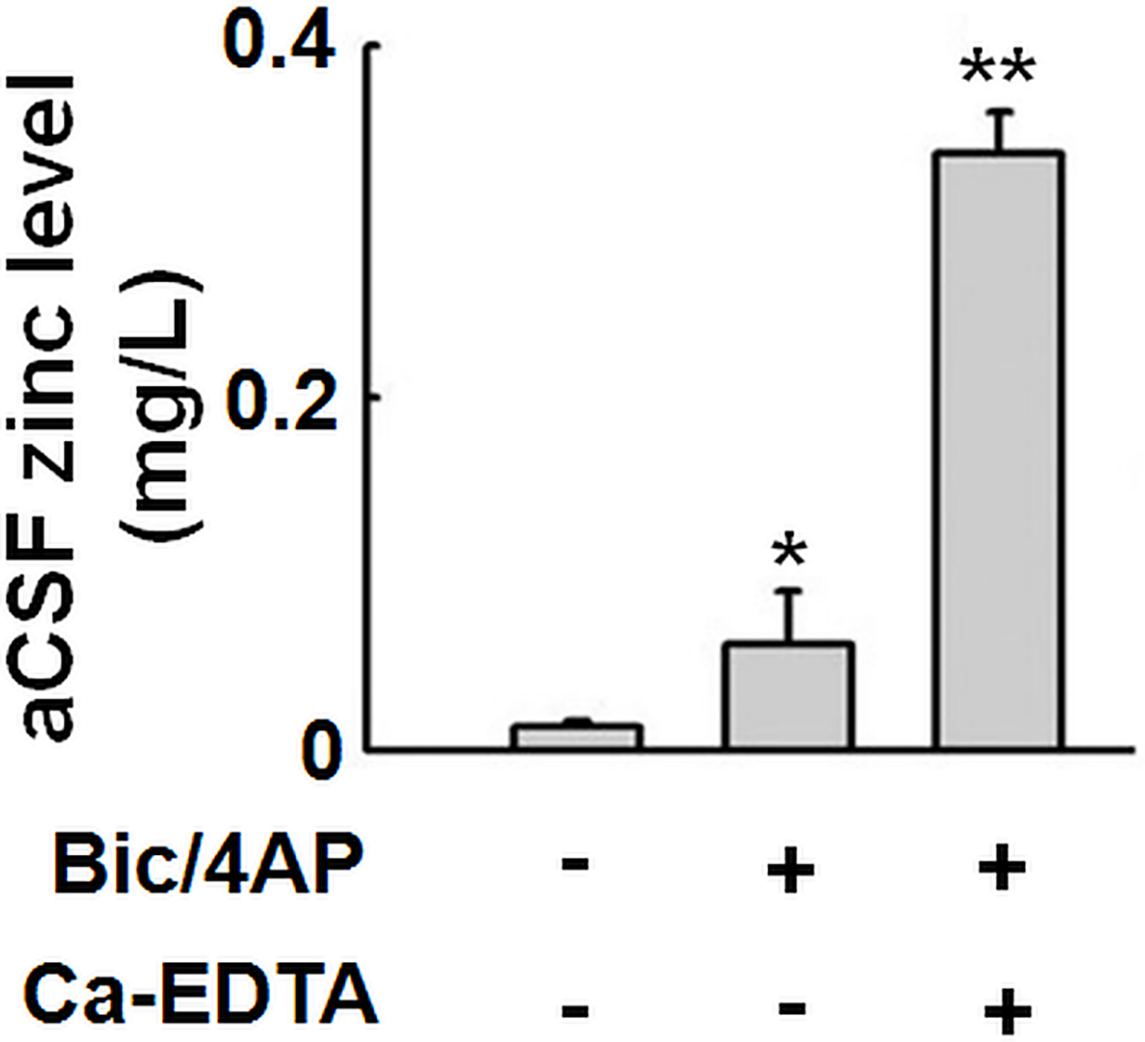
Increased synaptic activity induces zinc release in rat hippocampal slices. Rat hippocampal slices were incubated with Bic/4-AP for 3 h, with or without the pre-treatment of Ca-EDTA (1 mM) for 30 min. Zinc levels in aCSF were detected by atomic absorption spectrophotometer after incubation. * p<0.*05*, ** p<0.*01* versus untreated control slices. (n = 3).

During synaptic transmission of zinc-containing neurons, zinc is released from pre-synaptic vesicles into synaptic cleft, thus modulates the function of post-synaptic receptors or gets into post-synaptic neurons through zinc-permeable channels [22]. To explore whether synaptically released zinc also play a role in the anti-apoptosis effect of synaptic activity, we first tried to confirm zinc release during synaptic transmission in our experiment system. Hippocampus is the brain area which has the highest level of chetable zinc, that is, zinc in synaptic vesicles [23]. As shown in Fig 4, increased synaptic activity by Bic/4-AP treatment resulted in an increased level of zinc in aCSF, indicating zinc release from the cultured slices when synaptic activity was elevated. Pre-incubation with the cell-impermeable zinc chelator Ca-EDTA [24], which captures all the released zinc to avoid its entry into post-synaptic neurons or uptake into pre-synaptic neurons or glia, led to a dramatic increase in zinc in aCSF, confirming that a large amount of zinc ions were released during synaptic activity induced by Bic/4-AP in cultured hippocampal slices.

**Fig 5:**
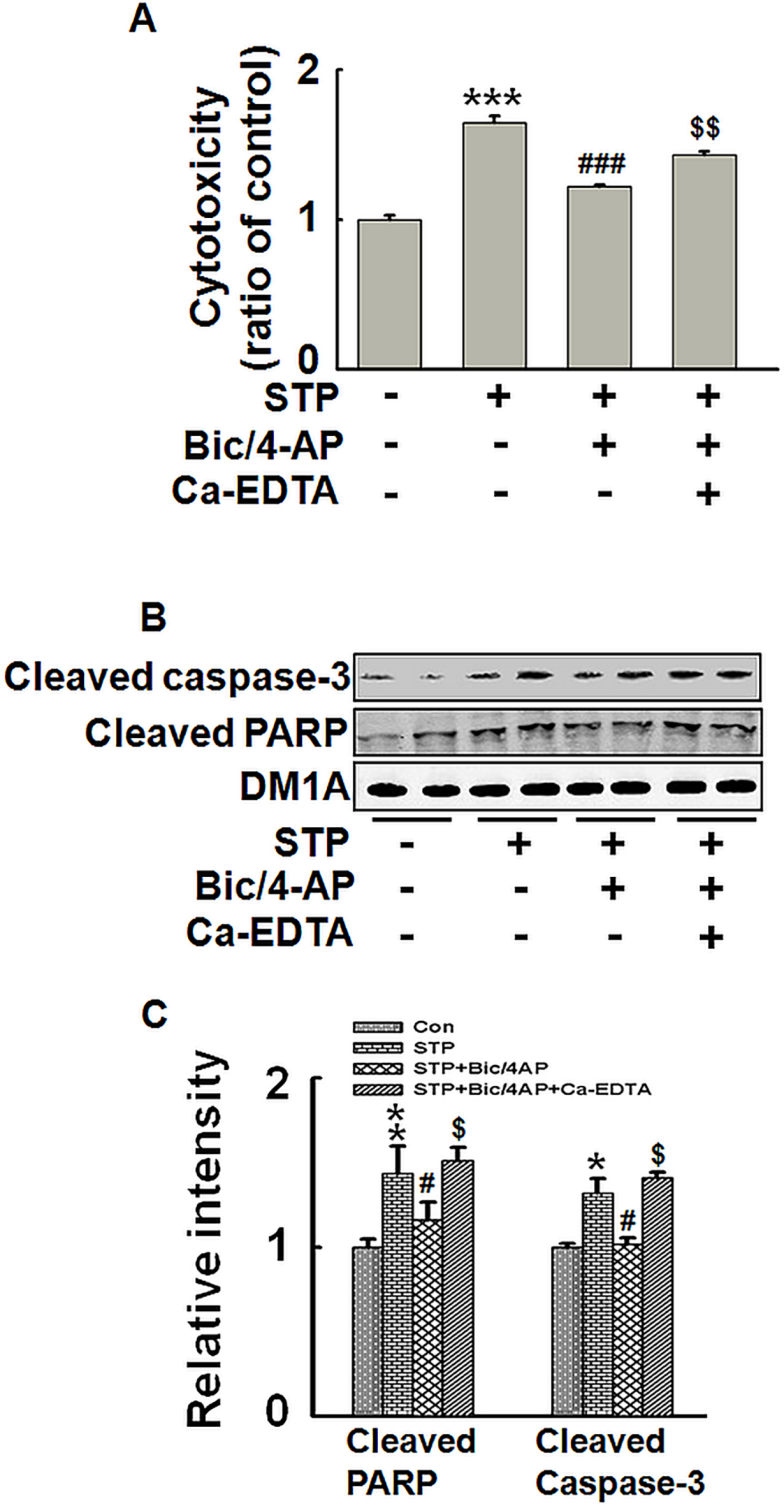
Activity-dependent anti-apoptotic effect is mediated by synaptically released zinc in rat hippocampal slices. Rat hippocampal slices were incubated with or without Ca-EDTA (1 mM) for 30 min. Then slices were treated with or without Bic (50 μM)/4-AP (250 μM) for 2.5 h. During the whole process, the slices were incubated with STP (1 μM, 3 h). (A) LDH levels in the aCSF. (B) Apoptosis related proteins were detected by Western blotting. (C) Quantitative analysis of blots in (B). * p<0.*05*,** p<0.*01*,*** p<0.*001* versus untreated control slices. *# p<0*.*05*, *### p<0*.*001* versus STP-treated slices. *$ p<0*.*05*, *$ $ p<0*.*01* versus STP/Bic/4-AP-treated slices. (n = 3).

Next, we explored the effect of synaptically released zinc on cell apoptosis in STP-treated hippocampal slices by using Ca-EDTA as the extracellular zinc chelator. Compared to STP-treated slices, increased synaptic activity (Bic/4-AP) prevented the cells from cytotoxicity as shown by LDH release. But this protective effect was not observed when synaptically released zinc was chelated (Fig 5A). This observation was further confirmed by the detection of apoptosis-related proteins. Two typical apoptosis indicators including cleaved caspase-3 and cleaved PARP were increased upon STP treatment, but the effect was reversed by application of Bic/4-AP; while Ca-EDTA prevented the protective anti-apoptotic effect of Bic/4-AP (Fig 5B and 5C). Thus, synaptic activity-induced anti-apoptotic effect is mediated by synaptically released zinc in rat hippocampal slices.

### Synaptically released zinc is required for the activity- dependent anti-apoptotic effect in cultured hippocampal neurons

**Fig 6:**
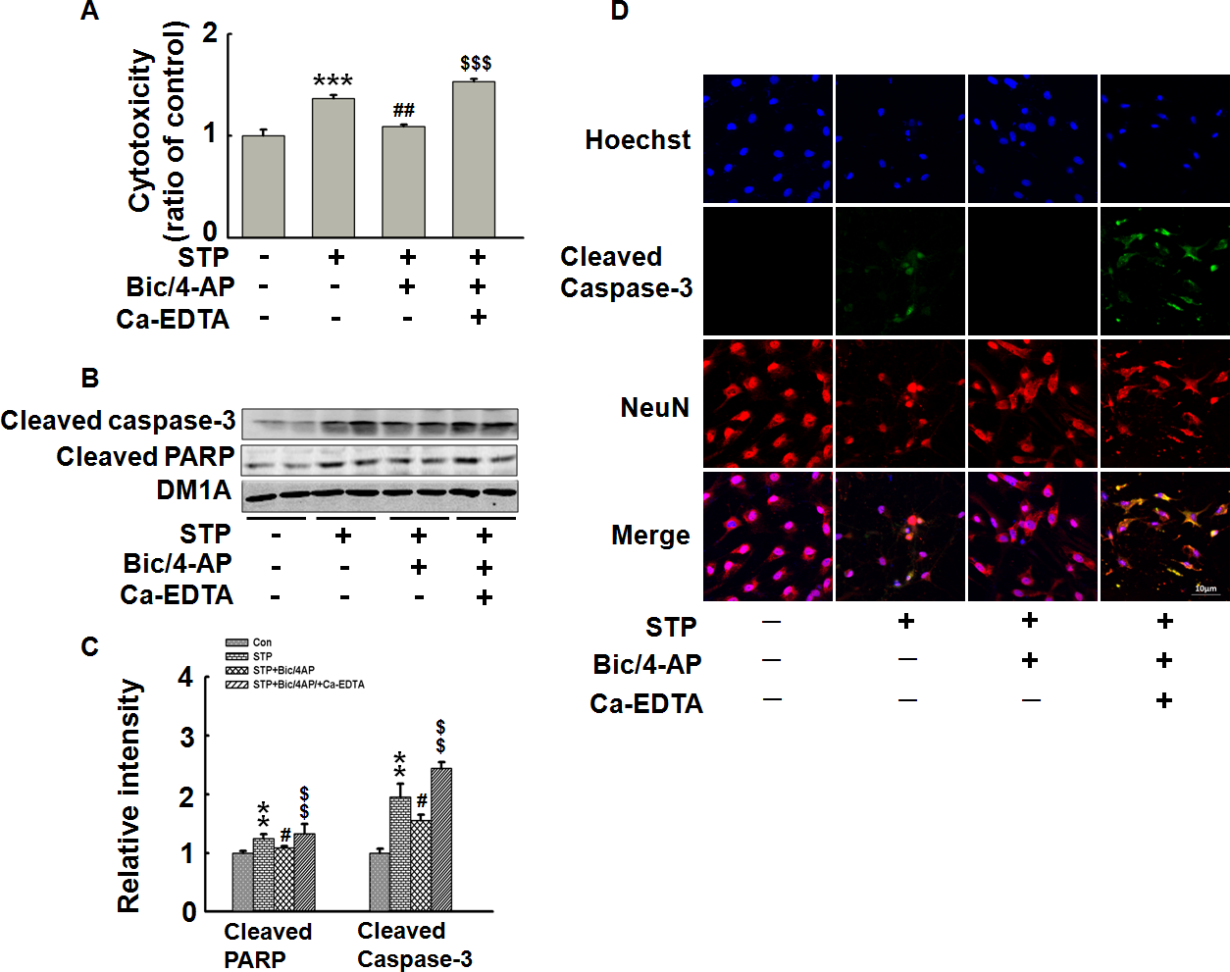
Synaptic activity-induced anti-apoptosis effect is mediated by synaptically released zinc in hippocampal neurons. Cultured hippocampal neurons were incubated with or without Ca-EDTA (1 mM) for 30 min. Then neurons were treated with STP (100 nM) for 24 h, with or without the pre-treatment of Bic/4-AP for 16 h. (A) Cytotoxicity was detected by LDH level assay. (B) Apoptosis related proteins were detected by Western blotting. (C) Quantitative analysis of blots in (B). ** p<0.*01*, *** p<0.*001* versus untreated control cells. *# p<0*.*05*, *## p<0*.*01* versus STP-treated cells. *$ $ p<0*.*01*, *$ $ $ p<0*.*001* versus STP/Bic/4-AP-treated cells. (D) Hippocampal neurons were fixed by 4% paraformaldehyde after incubation and immunostained with antibody against cleaved caspase-3 and NeuN. Scale bar = 10 μm. (n = 4).

To further confirm the above results, we repeated experiments in cultured primary hippocampal neurons. Cultured hippocampal neurons were incubated with or without Ca-EDTA (1mM) for 30 min. Then neurons were treated with STP (100 nM) for 24 h, with or without the pre-treatment of Bic/4-AP for 16 h. Similar results were observed in LDH cytotoxicity assay (Fig 6A) and Western blotting of apoptosis-related proteins (Fig 6B and 6C). Inmmunofluorescence staining of the neurons showed that STP induced significant cell apoptosis (positive staining of cleaved caspase-3), which was prevented by Bic/4-AP induced synaptic activity; and this protective effect was abolished when synaptically released zinc was captured by Ca-EDTA (Fig 6D). These results show that activity-dependent anti-apoptotic effect is mediated by synaptically released zinc in hippocampal neurons.

## Discussion

As an essential trace element in the body, zinc plays important roles in lots of functions in the brain, including neuronal development, neurogenesis, learning and memory [25–26]. Cytosolic free zinc concentration is very low in picomolar range; the major forms of zinc in neurons are zinc bound with metallothioneins and chetable (exchangeable) zinc in presynaptic vesicles [27]. Zinc-containing vesicles are enriched in the presynaptic terminals of glutamatergic neurons in brain areas such as hippocampus and amygdala [23]. These neurons are also referred to as gluzinergic neurons. When these neurons are excited, glutamate and zinc are released into synaptic cleft at the same time; and zinc acts as a neuromodulator to regulate the activity of glutamate receptors. Synaptically released zinc from gluzinergic neurons is involved in multiple events such as LTP formation and cognitive function [28–30]. Previous studies showed that synaptic activity of gluzinergic neurons can enhance the resistance of neurons to apoptosis, via multiple mechanisms including enhanced antioxidant defenses [5], transcriptional suppression of key components of the core apoptotic machinery [3], and upregulation of pro-survival gene expression [6, 31–32]. However, whether or not synaptically released zinc also plays a role in the anti-apoptotic effect of synaptic activity has not been elucidated. In the present study, we used two experimental models to show a crucial role for the released zinc in mediating the anti-apoptotic effect of synaptic activity in gluzinergic neurons.

We first used cultured hippocampal slices as an experimental model. Synaptic connections are more abundant in brain slices compared to that in cultured neurons. Thus, cultured brain slices can better mimic the *in vivo* situation. Hippocampus was selected because this area has a high density of gluzinergic neurons [9,10]. Bic/4-AP incubation is a classical strategy to induce synchronized bursts of action potentials, thus enhance synaptic activity. Increased synaptic activity upon Bic/4-AP incubation has been identified in several studies from different groups by using microelectrode array recordings [6], measurement of calcium influx [33] and detection of neuronal activity-induced gene expression [34]; Bic/4-AP treatment is also widely used in the studies to explore the anti-apoptotic effect of glutamatergic neuronal activity [3, 31]. In our previous study, we used Bic/4-AP or glutamate treatment in cultured hippocampal slices to enhance glutamatergic neuronal activity, the effect of these two treatments on zinc release was identified and Bic/4-AP treatment showed a better performance to induce zinc release [17]. In cultured hippocampal slices, we found that a commonly used apoptosis inducer—STP caused concentration-dependent cytotoxicity as detected by LDH release. The apoptotic cell death induced by STP was almost reversed by increased synaptic activity when the slices were pre-incubated with Bic/4-AP. Decreased protein levels of total caspase-3 and PARP confirmed cell apoptosis in STP treated slices, since decrease of these protein levels in short time (3 h) indicated the cleavage of caspase-3 and PARP. When synaptic activity was increased by Bic/4-AP, activation of caspase-3 was also reversed. Thus, in cultured hippocampal slices, our results confirmed the anti-apoptotic effect of enhanced synaptic activity.

Next, we confirmed the anti-apoptotic effect of zinc. It has been suspected that zinc concentration can reach up to 300 μM in the synaptic cleft upon activation of zinc-release neurons [12]. We pre-incubated the STP treated brain slices with 50 μM or 200 μM ZnSO_4_ and found that exogenous zinc in both concentrations effectively protected the cells from STP-induced apoptotic cytotoxicity. We further confirmed that in our experimental model, zinc ions were released from the slices when synaptic activity was increased. Ca-EDTA is a cell-impermeable zinc chelator, which can capture all the released zinc ions from pre-synaptic vesicles during bursts of action potentials. In our experiment, we found that zinc level was slightly increased in aCSF upon Bic/4-AP treatment. When Ca-EDTA was applied, zinc level in aCSF was significantly increased. These results identify that there is a large amount of zinc ions released outside of the neurons when synaptic activity is increased by Bic/4-AP. Ca-EDTA prevents the uptake of extracellular zinc by glia or pre-synaptic terminals, or zinc entry into the post-synaptic neurons, thus keeping zinc ions in the aCSF. Therefore, zinc has anti-apoptotic effect, and zinc ions are released from activated neurons during bursts of action potentials.

We further explored the role of synaptically released zinc in anti-apoptotic effect of synaptic activity. The results showed that when the synaptically released zinc was chelated by Ca-EDTA, synaptic activity failed to show protective effect in cultured slices. The same results were observed in cultured primary hippocampal neurons. Compared with cultured brain slices, Bic/4-AP treatment to cultured neurons induced stronger prevention against STP-induced cytotoxicity, and Ca-EDTA pre-incubation almost completely blocked this effect. The better effects of both Bic/4-AP and Ca-EDTA may be due to the longer incubation of the neurons with these reagents. In a summary, these results indicate that synaptically released zinc mediates the anti-apoptotic effect of synaptic activity in gluzinergic neurons.

The mechanism of synaptic zinc-mediated anti-apoptotic effect is still unknown. Zinc ions may be involved in regulating the transcription of apoptosis-related proteins, or suppress apoptosis through inhibiting PP2A [17], which has been reported to promote apoptosis through complex pathways [35–36]. Further work is needed to clarify the underlying molecular details.

## Supporting information

S1 File NC3Rs ARRIVE guidelines check list. (PDF)

S2 File Original data for the statistic analysis. (DOC)
