# Supplementary material for: Zinc mediates the neuronal activity–dependent anti-apoptotic effect
Source: PLoS One. 2017 Aug 7;12(8):e0182150. doi: 10.1371/journal.pone.0182150 (PMC5546700; doi:10.1371/journal.pone.0182150)
Supplement: S2 File — (DOC) [file pone.0182150.s002.doc]

Fig1
con	0.239	0.249	0.248	0.256	0.242	0.224	
0.2um	0.298	0.316	0.286	0.242	0.273	0.26	
0.5um	0.332	0.367	0.312	0.293	0.282	0.302	
0.8um	0.236	0.24	0.229	0.429	0.406	0.399	
1um	0.444	0.471	0.447	0.376	0.4	0.402	
2um	0.695	0.647	0.732	0.338	0.353	0.343	
anoxia	0.895	0.897	0.921				
positive control	2.183	2.179	2.19				

Descriptives	
VAR00002								
	N	Mean	Std. Deviation	Std. Error	95% Confidence Interval for Mean	Minimum	Maximum	
					Lower Bound	Upper Bound			
1	6	.2430	.01103	.00450	.2314	.2546	.22	.26	
2	6	.2792	.02663	.01087	.2512	.3071	.24	.32	
3	6	.3147	.03080	.01257	.2823	.3470	.28	.37	
4	6	.3232	.09715	.03966	.2212	.4251	.23	.43	
5	6	.4233	.03605	.01472	.3855	.4612	.38	.47	
6	6	.5180	.19184	.07832	.3167	.7193	.34	.73	
7	3	.9043	.01447	.00835	.8684	.9403	.90	.92	
Total	39	.3928	.19259	.03084	.3304	.4553	.22	.92	

Multiple Comparisons	
VAR00002
LSD						
(I) VAR00001	(J) VAR00001	Mean Difference (I-J)	Std. Error	Sig.	95% Confidence Interval	
					Lower Bound	Upper Bound	
1	2	-.03617	.05073	.481	-.1395	.0672	
	3	-.07167	.05073	.167	-.1750	.0317	
	4	-.08017	.05073	.124	-.1835	.0232	
	5	-.18033*	.05073	.001	-.2837	-.0770	
	6	-.27500*	.05073	.000	-.3783	-.1717	
	7	-.66133*	.06213	.000	-.7879	-.5348	
2	1	.03617	.05073	.481	-.0672	.1395	
	3	-.03550	.05073	.489	-.1388	.0678	
	4	-.04400	.05073	.392	-.1473	.0593	
	5	-.14417*	.05073	.008	-.2475	-.0408	
	6	-.23883*	.05073	.000	-.3422	-.1355	
	7	-.62517*	.06213	.000	-.7517	-.4986	
3	1	.07167	.05073	.167	-.0317	.1750	
	2	.03550	.05073	.489	-.0678	.1388	
	4	-.00850	.05073	.868	-.1118	.0948	
	5	-.10867*	.05073	.040	-.2120	-.0053	
	6	-.20333*	.05073	.000	-.3067	-.1000	
	7	-.58967*	.06213	.000	-.7162	-.4631	
4	1	.08017	.05073	.124	-.0232	.1835	
	2	.04400	.05073	.392	-.0593	.1473	
	3	.00850	.05073	.868	-.0948	.1118	
	5	-.10017	.05073	.057	-.2035	.0032	
	6	-.19483*	.05073	.001	-.2982	-.0915	
	7	-.58117*	.06213	.000	-.7077	-.4546	
5	1	.18033*	.05073	.001	.0770	.2837	
	2	.14417*	.05073	.008	.0408	.2475	
	3	.10867*	.05073	.040	.0053	.2120	
	4	.10017	.05073	.057	-.0032	.2035	
	6	-.09467	.05073	.071	-.1980	.0087	
	7	-.48100*	.06213	.000	-.6075	-.3545	
6	1	.27500*	.05073	.000	.1717	.3783	
	2	.23883*	.05073	.000	.1355	.3422	
	3	.20333*	.05073	.000	.1000	.3067	
	4	.19483*	.05073	.001	.0915	.2982	
	5	.09467	.05073	.071	-.0087	.1980	
	7	-.38633*	.06213	.000	-.5129	-.2598	
7	1	.66133*	.06213	.000	.5348	.7879	
	2	.62517*	.06213	.000	.4986	.7517	
	3	.58967*	.06213	.000	.4631	.7162	
	4	.58117*	.06213	.000	.4546	.7077	
	5	.48100*	.06213	.000	.3545	.6075	
	6	.38633*	.06213	.000	.2598	.5129	
*. The mean difference is significant at the 0.05 level.

		
Fig2A
con	0.226	0.235	0.231	0.23	0.244	0.247	0.353	0.348	0.38	
stp	0.568	0.361	0.393	0.459						
bic	0.259	0.234	0.253	0.335	0.344	0.323	0.291	0.268	0.28	
bic+stp	0.222	0.237	0.225	0.243	0.247	0.265	0.195	0.18	0.213	

Descriptives	
VAR00002								
	N	Mean	Std. Deviation	Std. Error	95% Confidence Interval for Mean	Minimum	Maximum	
					Lower Bound	Upper Bound			
1	9	.2771	.06335	.02112	.2284	.3258	.23	.38	
2	4	.4453	.09144	.04572	.2997	.5908	.36	.57	
3	9	.2874	.03877	.01292	.2576	.3172	.23	.34	
4	9	.2252	.02653	.00884	.2048	.2456	.18	.26	
Total	31	.2867	.08371	.01504	.2560	.3174	.18	.57	


Multiple Comparisons	
VAR00002
LSD						
(I) VAR00001	(J) VAR00001	Mean Difference (I-J)	Std. Error	Sig.	95% Confidence Interval	
					Lower Bound	Upper Bound	
1	2	-.16814*	.03164	.000	-.2331	-.1032	
	3	-.01033	.02482	.680	-.0613	.0406	
	4	.05189*	.02482	.046	.0010	.1028	
2	1	.16814*	.03164	.000	.1032	.2331	
	3	.15781*	.03164	.000	.0929	.2227	
	4	.22003*	.03164	.000	.1551	.2849	
3	1	.01033	.02482	.680	-.0406	.0613	
	2	-.15781*	.03164	.000	-.2227	-.0929	
	4	.06222*	.02482	.018	.0113	.1131	
4	1	-.05189*	.02482	.046	-.1028	-.0010	
	2	-.22003*	.03164	.000	-.2849	-.1551	
	3	-.06222*	.02482	.018	-.1131	-.0113	
*. The mean difference is significant at the 0.05 level.		


Fig2C
Caspase3		PARP		cytc		
	I.I.(K Counts)		I.I.(K Counts)		I.I.(K Counts)	
con	1.53	con	3.67	con	4.74	
	1.58		4.11		4.35	
	1.65		3.56		4.31	
STP	1.42	STP	3.05	STP	5.18	
	1.42		2.94		5.12	
	1.5		3.37		4.66	
bic	1.75	bic	3.88	bic	3.84	
	1.67		3.9		3.64	
	1.72		4.25		4	
bic+STP	1.61	bic+STP	4.01	bic+STP	4.44	
	1.7		4.56		4.27	
	1.56		4.59		4.65	
Caspase3
Descriptives	
VAR00002								
	N	Mean	Std. Deviation	Std. Error	95% Confidence Interval for Mean	Minimum	Maximum	
					Lower Bound	Upper Bound			
1	3	1.0000	.03799	.02193	.9056	1.0944	.96	1.04	
2	3	.9118	.02911	.01681	.8395	.9841	.89	.95	
3	3	1.0798	.02547	.01471	1.0166	1.1431	1.05	1.10	
4	3	1.0231	.04471	.02582	.9120	1.1342	.98	1.07	
Total	12	1.0037	.06993	.02019	.9592	1.0481	.89	1.10	

Multiple Comparisons	
VAR00002
LSD						
(I) VAR00001	(J) VAR00001	Mean Difference (I-J)	Std. Error	Sig.	95% Confidence Interval	
					Lower Bound	Upper Bound	
1	2	.08824*	.02869	.015	.0221	.1544	
	3	-.07983*	.02869	.024	-.1460	-.0137	
	4	-.02311	.02869	.444	-.0893	.0431	
2	1	-.08824*	.02869	.015	-.1544	-.0221	
	3	-.16807*	.02869	.000	-.2342	-.1019	
	4	-.11134*	.02869	.005	-.1775	-.0452	
3	1	.07983*	.02869	.024	.0137	.1460	
	2	.16807*	.02869	.000	.1019	.2342	
	4	.05672	.02869	.083	-.0094	.1229	
4	1	.02311	.02869	.444	-.0431	.0893	
	2	.11134*	.02869	.005	.0452	.1775	
	3	-.05672	.02869	.083	-.1229	.0094	
*. The mean difference is significant at the 0.05 level.		
PARP
Descriptives	
VAR00002								
	N	Mean	Std. Deviation	Std. Error	95% Confidence Interval for Mean	Minimum	Maximum	
					Lower Bound	Upper Bound			
1	3	1.0000	.07699	.04445	.8087	1.1913	.94	1.09	
2	3	.8254	.05910	.03412	.6786	.9722	.78	.89	
3	3	1.0608	.05505	.03178	.9241	1.1976	1.03	1.12	
4	3	1.1605	.08639	.04988	.9459	1.3751	1.06	1.21	
Total	12	1.0117	.14079	.04064	.9222	1.1011	.78	1.21	

Multiple Comparisons	
VAR00002
LSD						
(I) VAR00001	(J) VAR00001	Mean Difference (I-J)	Std. Error	Sig.	95% Confidence Interval	
					Lower Bound	Upper Bound	
1	2	.17460*	.05761	.016	.0418	.3075	
	3	-.06085	.05761	.322	-.1937	.0720	
	4	-.16049*	.05761	.024	-.2933	-.0276	
2	1	-.17460*	.05761	.016	-.3075	-.0418	
	3	-.23545*	.05761	.004	-.3683	-.1026	
	4	-.33510*	.05761	.000	-.4679	-.2022	
3	1	.06085	.05761	.322	-.0720	.1937	
	2	.23545*	.05761	.004	.1026	.3683	
	4	-.09965	.05761	.122	-.2325	.0332	
4	1	.16049*	.05761	.024	.0276	.2933	
	2	.33510*	.05761	.000	.2022	.4679	
	3	.09965	.05761	.122	-.0332	.2325	
*. The mean difference is significant at the 0.05 level.		
Cyt-C

Descriptives	
VAR00002								
	N	Mean	Std. Deviation	Std. Error	95% Confidence Interval for Mean	Minimum	Maximum	
					Lower Bound	Upper Bound			
1	3	1.0000	.05318	.03071	.8679	1.1321	.96	1.06	
2	3	1.1164	.06369	.03677	.9582	1.2746	1.04	1.16	
3	3	.8567	.04038	.02331	.7564	.9570	.81	.90	
4	3	.9970	.04262	.02460	.8912	1.1029	.96	1.04	
Total	12	.9925	.10543	.03044	.9255	1.0595	.81	1.16	


Multiple Comparisons	
VAR00002
LSD						
(I) VAR00001	(J) VAR00001	Mean Difference (I-J)	Std. Error	Sig.	95% Confidence Interval	
					Lower Bound	Upper Bound	
1	2	-.11642*	.04150	.023	-.2121	-.0207	
	3	.14328*	.04150	.009	.0476	.2390	
	4	.00299	.04150	.944	-.0927	.0987	
2	1	.11642*	.04150	.023	.0207	.2121	
	3	.25970*	.04150	.000	.1640	.3554	
	4	.11940*	.04150	.021	.0237	.2151	
3	1	-.14328*	.04150	.009	-.2390	-.0476	
	2	-.25970*	.04150	.000	-.3554	-.1640	
	4	-.14030*	.04150	.010	-.2360	-.0446	
4	1	-.00299	.04150	.944	-.0987	.0927	
	2	-.11940*	.04150	.021	-.2151	-.0237	
	3	.14030*	.04150	.010	.0446	.2360	
*. The mean difference is significant at the 0.05 level.		

Fig3A
CON	0.195	0.18	0.196				
STP	0.327	0.418	0.309	0.313	0.241		
stp+50um	0.189	0.241	0.173	0.241	0.187	0.225	
stp+200um	0.143	0.17	0.149	0.274	0.271	0.308	
Descriptives	
VAR00001								
	N	Mean	Std. Deviation	Std. Error	95% Confidence Interval for Mean	Minimum	Maximum	
					Lower Bound	Upper Bound			
1	3	.1903	.00896	.00517	.1681	.2126	.18	.20	
2	5	.3216	.06335	.02833	.2429	.4003	.24	.42	
3	6	.2093	.02994	.01222	.1779	.2408	.17	.24	
4	6	.2192	.07311	.02985	.1424	.2959	.14	.31	
Total	20	.2375	.07117	.01591	.2042	.2708	.14	.42	
Multiple Comparisons	
VAR00001
LSD						
(I) VAR00002	(J) VAR00002	Mean Difference (I-J)	Std. Error	Sig.	95% Confidence Interval	
					Lower Bound	Upper Bound	
1	2	-.13127*	.03976	.005	-.2156	-.0470	
	3	-.01900	.03850	.628	-.1006	.0626	
	4	-.02883	.03850	.465	-.1104	.0528	
2	1	.13127*	.03976	.005	.0470	.2156	
	3	.11227*	.03297	.004	.0424	.1822	
	4	.10243*	.03297	.007	.0325	.1723	
3	1	.01900	.03850	.628	-.0626	.1006	
	2	-.11227*	.03297	.004	-.1822	-.0424	
	4	-.00983	.03143	.758	-.0765	.0568	
4	1	.02883	.03850	.465	-.0528	.1104	
	2	-.10243*	.03297	.007	-.1723	-.0325	
	3	.00983	.03143	.758	-.0568	.0765	
*. The mean difference is significant at the 0.05 level.		

Fig3B 
2.5 h
CON	0.994	1.016	1.067	0.965	1.043	1.075	1.074	1.07	1.108	
50uMZnSO4	0.99	0.896	1.138	1.067	1.056	1.101	1.086	1.154	1.089	
200uMZnSO4	0.986	1.174	0.929	0.941	0.948	0.945	0.959	1.207	0.995	

Table Analyzed	Data 1	¡¡	¡¡	¡¡	¡¡	
¡¡	¡¡	¡¡	¡¡	¡¡	¡¡	
One-way analysis of variance	¡¡	¡¡	¡¡	¡¡	¡¡	
P value	0.3544	¡¡	¡¡	¡¡	¡¡	
P value summary	ns	¡¡	¡¡	¡¡	¡¡	
Are means signif. different? (P < 0.05)	No	¡¡	¡¡	¡¡	¡¡	
Number of groups	3	¡¡	¡¡	¡¡	¡¡	
F	1.084	¡¡	¡¡	¡¡	¡¡	
R squared	0.08282	¡¡	¡¡	¡¡	¡¡	
¡¡	¡¡	¡¡	¡¡	¡¡	¡¡	
Bartlett's test for equal variances	¡¡	¡¡	¡¡	¡¡	¡¡	
Bartlett's statistic (corrected)	4.772	¡¡	¡¡	¡¡	¡¡	
P value	0.092	¡¡	¡¡	¡¡	¡¡	
P value summary	ns	¡¡	¡¡	¡¡	¡¡	
Do the variances differ signif. (P < 0.05)	No	¡¡	¡¡	¡¡	¡¡	
¡¡	¡¡	¡¡	¡¡	¡¡	¡¡	
ANOVA Table	SS	df	MS	¡¡	¡¡	
Treatment (between columns)	0.01399	2	0.006997	¡¡	¡¡	
Residual (within columns)	0.155	24	0.006458	¡¡	¡¡	
Total	0.169	26	¡¡	¡¡	¡¡	
¡¡	¡¡	¡¡	¡¡	¡¡	¡¡	
Tukey's Multiple Comparison Test	Mean Diff.	q	Significant? P < 0.05?	Summary	95% CI of diff	
0 uM ZnSO4 vs 50 uM ZnSO4	-0.01833	0.6844	No	ns	-0.1129 to 0.07628	
0 uM ZnSO4 vs 200 uM ZnSO4	0.03644	1.361	No	ns	-0.05816 to 0.1311	
50 uM ZnSO4 vs 200 uM ZnSO4	0.05478	2.045	No	ns	-0.03983 to 0.1494	

5.5 h
CON	0.92907	0.980233	1.153488	1.093023	1.089535	0.933721	0.975581	0.909302	1.036047	
50uMZnSO4	0.888372	0.895349	0.961628	0.95	1.037209	0.983721	0.994186	0.947674	1.066279	
200uMZnSO4	0.99186	0.975581	1.031395	0.92907	0.884884	0.998837	0.984884	1.024419	1.082558	

Table Analyzed	Data 1	¡¡	¡¡	¡¡	¡¡	
¡¡	¡¡	¡¡	¡¡	¡¡	¡¡	
One-way analysis of variance	¡¡	¡¡	¡¡	¡¡	¡¡	
P value	0.4481	¡¡	¡¡	¡¡	¡¡	
P value summary	ns	¡¡	¡¡	¡¡	¡¡	
Are means signif. different? (P < 0.05)	No	¡¡	¡¡	¡¡	¡¡	
Number of groups	3	¡¡	¡¡	¡¡	¡¡	
F	0.8302	¡¡	¡¡	¡¡	¡¡	
R squared	0.06471	¡¡	¡¡	¡¡	¡¡	
¡¡	¡¡	¡¡	¡¡	¡¡	¡¡	
Bartlett's test for equal variances	¡¡	¡¡	¡¡	¡¡	¡¡	
Bartlett's statistic (corrected)	1.63	¡¡	¡¡	¡¡	¡¡	
P value	0.4427	¡¡	¡¡	¡¡	¡¡	
P value summary	ns	¡¡	¡¡	¡¡	¡¡	
Do the variances differ signif. (P < 0.05)	No	¡¡	¡¡	¡¡	¡¡	
¡¡	¡¡	¡¡	¡¡	¡¡	¡¡	
ANOVA Table	SS	df	MS	¡¡	¡¡	
Treatment (between columns)	0.007842	2	0.003921	¡¡	¡¡	
Residual (within columns)	0.1134	24	0.004723	¡¡	¡¡	
Total	0.1212	26	¡¡	¡¡	¡¡	
¡¡	¡¡	¡¡	¡¡	¡¡	¡¡	
Tukey's Multiple Comparison Test	Mean Diff.	q	Significant? P < 0.05?	Summary	95% CI of diff	
0 uM ZnSO4 vs 50 uM ZnSO4	0.04173	1.822	No	ns	-0.03918 to 0.1226	
0 uM ZnSO4 vs 200 uM ZnSO4	0.02183	0.9531	No	ns	-0.05908 to 0.1027	
50 uM ZnSO4 vs 200 uM ZnSO4	-0.0199	0.8685	No	ns	-0.1008 to 0.06101	

Fig5A
con1	0.267	0.254	0.27	
con2	0.216	0.212	0.246	
con3	0.195	0.202	0.204	
stp1	0.252	0.251	0.262	
stp2	0.357	0.372	0.357	
stp3	0.333	0.318	0.344	
bic+stp1	0.248	0.266	0.257	
bic+stp2	0.264	0.279	0.263	
bic+stp3	0.256	0.246	0.258	
bic+stp+ca1	0.302	0.317	0.291	
bic+stp+ca2	0.28	0.277	0.283	
bic+stp+ca3	0.34	0.325	0.32	
Descriptives	
VAR00001								
	N	Mean	Std. Deviation	Std. Error	95% Confidence Interval for Mean	Minimum	Maximum	
					Lower Bound	Upper Bound			
1	9	.2296	.02959	.00986	.2068	.2523	.20	.27	
2	9	.3162	.04849	.01616	.2789	.3535	.25	.37	
3	9	.2597	.00994	.00331	.2520	.2673	.25	.28	
4	9	.3039	.02259	.00753	.2865	.3212	.28	.34	
Total	36	.2773	.04598	.00766	.2618	.2929	.20	.37	
Multiple Comparisons	
VAR00001
LSD						
(I) VAR00002	(J) VAR00002	Mean Difference (I-J)	Std. Error	Sig.	95% Confidence Interval	
					Lower Bound	Upper Bound	
1	2	-.08667*	.01460	.000	-.1164	-.0569	
	3	-.03011*	.01460	.047	-.0598	-.0004	
	4	-.07433*	.01460	.000	-.1041	-.0446	
2	1	.08667*	.01460	.000	.0569	.1164	
	3	.05656*	.01460	.000	.0268	.0863	
	4	.01233	.01460	.404	-.0174	.0421	
3	1	.03011*	.01460	.047	.0004	.0598	
	2	-.05656*	.01460	.000	-.0863	-.0268	
	4	-.04422*	.01460	.005	-.0740	-.0145	
4	1	.07433*	.01460	.000	.0446	.1041	
	2	-.01233	.01460	.404	-.0421	.0174	
	3	.04422*	.01460	.005	.0145	.0740	
*. The mean difference is significant at the 0.05 level.		

Fig6A
con	0.247	0.273	0.368	0.19	0.258	0.219	
atp	0.319	0.327	0.384	0.401	0.354	0.339	
STP+BIC	0.268	0.307	0.309	0.263	0.269	0.28	
STP+B+CA	0.396	0.43	0.426	0.368	0.364	0.398	

Descriptives	
VAR00001								
	N	Mean	Std. Deviation	Std. Error	95% Confidence Interval for Mean	Minimum	Maximum	
					Lower Bound	Upper Bound			
1	6	.2592	.06096	.02489	.1952	.3231	.19	.37	
2	6	.3540	.03252	.01328	.3199	.3881	.32	.40	
3	6	.2827	.02040	.00833	.2613	.3041	.26	.31	
4	6	.3970	.02779	.01135	.3678	.4262	.36	.43	
Total	24	.3232	.06680	.01364	.2950	.3514	.19	.43	
Multiple Comparisons	
VAR00001
LSD						
(I) VAR00002	(J) VAR00002	Mean Difference (I-J)	Std. Error	Sig.	95% Confidence Interval	
					Lower Bound	Upper Bound	
1	2	-.09483*	.02229	.000	-.1413	-.0483	
	3	-.02350	.02229	.304	-.0700	.0230	
	4	-.13783*	.02229	.000	-.1843	-.0913	
2	1	.09483*	.02229	.000	.0483	.1413	
	3	.07133*	.02229	.004	.0248	.1178	
	4	-.04300	.02229	.068	-.0895	.0035	
3	1	.02350	.02229	.304	-.0230	.0700	
	2	-.07133*	.02229	.004	-.1178	-.0248	
	4	-.11433*	.02229	.000	-.1608	-.0678	
4	1	.13783*	.02229	.000	.0913	.1843	
	2	.04300	.02229	.068	-.0035	.0895	
	3	.11433*	.02229	.000	.0678	.1608	
*. The mean difference is significant at the 0.05 level.		

Fig6C
Cleaved-PARP		
	1	2	
con	0.96	0.71	
	0.91	0.66	
stp	1.09	0.92	
	1.14	0.86	
bic+stp	1.01	0.75	
	1.05	0.72	
bic+stp+ca	1.39	0.99	
	1.14	0.79	

Descriptives	
VAR00002								
	N	Mean	Std. Deviation	Std. Error	95% Confidence Interval for Mean	Minimum	Maximum	
					Lower Bound	Upper Bound			
1	4	1.0000	.03694	.01847	.9412	1.0588	.96	1.04	
2	4	1.2459	.07453	.03726	1.1273	1.3645	1.17	1.34	
3	4	1.0873	.02996	.01498	1.0396	1.1350	1.05	1.12	
4	4	1.3261	.16457	.08229	1.0642	1.5880	1.15	1.49	
Total	16	1.1648	.15658	.03914	1.0814	1.2483	.96	1.49	


Multiple Comparisons	
VAR00002
LSD						
(I) VAR00001	(J) VAR00001	Mean Difference (I-J)	Std. Error	Sig.	95% Confidence Interval	
					Lower Bound	Upper Bound	
1	2	-.24589*	.06605	.003	-.3898	-.1020	
	3	-.08730	.06605	.211	-.2312	.0566	
	4	-.32611*	.06605	.000	-.4700	-.1822	
2	1	.24589*	.06605	.003	.1020	.3898	
	3	.15859*	.06605	.033	.0147	.3025	
	4	-.08021	.06605	.248	-.2241	.0637	
3	1	.08730	.06605	.211	-.0566	.2312	
	2	-.15859*	.06605	.033	-.3025	-.0147	
	4	-.23881*	.06605	.004	-.3827	-.0949	
4	1	.32611*	.06605	.000	.1822	.4700	
	2	.08021	.06605	.248	-.0637	.2241	
	3	.23881*	.06605	.004	.0949	.3827	
*. The mean difference is significant at the 0.05 level.		
